# Synchronous clonally related anaplastic large cell lymphoma and malignant histiocytosis

**DOI:** 10.1186/s13000-025-01597-3

**Published:** 2025-01-14

**Authors:** Mirvate Harb, Tom Abrassart, Laurent Dewispeleare, Pierre Sidon, Natacha Dirckx, Anne-laure Trepant, Julie Castiaux, Pierre Heimann, Jean-Francois Emile, Hussein Farhat

**Affiliations:** 1 Laboratoire Hospitalier Universitaire de Bruxelles - Universitair Laboratorium Brussel, Université Libre de Bruxelles LHUB-ULB, Brussels, Belgium; 2 Erasme Hospital, Brussels, Belgium; 3 Jules Bordet Institute, Hematology, Brussels, Belgium; 4 Paris-Saclay University, EA4340-BECCOH, Versailles SQY University, Assistance Publique–Hôpitaux de Paris [AP-HP], Ambroise-Paré Hospital, Smart Imaging, Service de Pathologie, Paris, France

**Keywords:** Secondary malignant histiocytosis, Synchronous malignant histiocytosis, Anaplastic large cell lymphoma, Case report

## Abstract

**Background:**

Synchronous malignant histiocytoses are rare conditions that occur concurrently with another hematologic neoplasm. Most reported cases are associated with B-cell lymphoproliferative disorders, while associations with T-cell hemopathies are less common. These two diseases may share mutations and/or cytogenetic anomalies, which can lead to malignant proliferations. In such cases, the term “secondary malignant histiocytosis” can be applied.

**Case description:**

A 26-year-old patient was diagnosed with anaplastic lymphoma kinase negative anaplastic large cell lymphoma [ALK-ALCL] associated with synchronous malignant histiocytosis. Neoplastic cells were distinguished by the exclusivity of the rearrangement of TCR genes within the lymphoma cells, whereas mutations in the *KRAS* and *TP53* genes affected mono-histiocytic cells. However, these two cells populations shared common chromosomal abnormalities. First line treatment protocol included Brentuximab vedotin, cyclophosphamide, doxorubicin, and methylprednisolone. Despite a partial clinical and biological response after cycle 1 of treatment, the patient was refractory at the end of cycle 2. Patient died in the intensive care unit from a multiple-organ failure related to lymphohistiocytic hemophagocytosis.

**Conclusion:**

This case represents the first documented instance of synchronous malignant histiocytosis associated with anaplastic large cell lymphoma. Notably, the uniqueness of this case lies in the absence of TCR rearrangement in the histiocytic cells, despite the presence of shared chromosomal abnormalities with the lymphomatous cells indicating a common origin for both neoplastic proliferations. Considering the rarity of such occurrences, the use of histiocytosis targeted therapy alongside conventional lymphoma treatment warrants consideration in such a context.

## Background

Synchronous malignant histiocytoses are rare conditions that occur concurrently with another hematologic neoplasm [1]. Most of such reported cases were associated with B-cell lymphoproliferative disorders including follicular lymphomas, in which they shared the IgH-BCL2 rearrangement [2]. Less frequently, they were associated with T-cell malignant disorders, mostly of immature nature, such as T lymphoblastic lymphoma / leukemia [3]. In such instances, the monoclonal rearrangement of the TCR gene represented the main genetic anomaly shared by both types of neoplastic cells. In the presence of common cytogenetic/ molecular clonal aberration in both populations, the term “secondary malignant histiocytosis” can be applied.

We report here the first case of anaplastic lymphoma kinase negative anaplastic large cell lymphoma [ALK-ALCL] associated with synchronous malignant histiocytosis where the histiocytic component lacked the TCR gene rearrangement found in the lymphoma cells.

## Case description

We report the case of a 26-year-old patient with unremarkable past medical history.

He presented with a two-weeks history of symptoms including weight loss of 5 kg, fever, and sweating. On physical examination, an upper extremity venous syndrome was noted, with palpation of a left supraclavicular lymph node. Hepatomegaly and splenomegaly of 2 centimeters below the costal margin were also noted.

Laboratory investigations were notable for the following results: hemoglobin 7.1 g/dL [reference values [RV]: 13–18 g/dL], MCV 86 fL [RV: 80–100 fL], platelets 21.000/mm³ [RV: 150.000-440.000/mm³], neutrophils 2870/mm³ [RV: 1500–6700/mm³], lymphocytes 560/mm³ [RV: 1200–3500/mm³], ferritine 1800 µg/L [RV: 30–300 µg/L], lactate dehydrogenase 10.497 UI/L [RV: 135–225 UI/L], alanine aminotransferase 57 U/L [RV: < 40U/L], aspartate aminotransferase 95 U/L [RV: < 40 U/L], total bilirubin 1.3 mg/dL [RV: < 1.2 mg/dL].

**Fig. 1:**
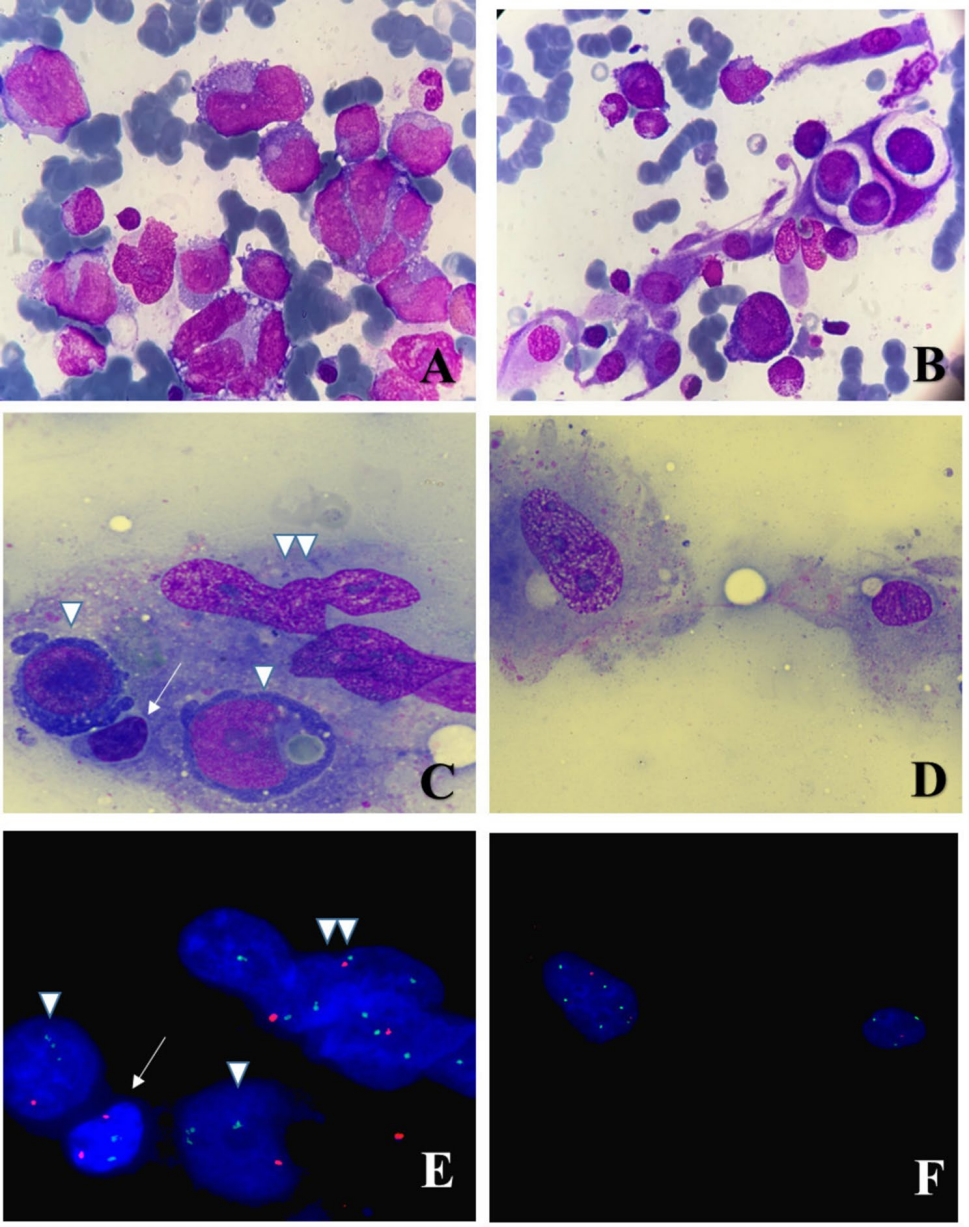
**A**: Cytological examination of the bone marrow aspiration showing massive infiltration by large pleiomorphic cells with some of them displaying the morphological features of “hallmark” cells that are typically associated with anaplastic large cell lymphomas [x1000, May Grunwald-Giemsa [MGG] stain]. **B**: Histiocytic neoplastic proliferation with some cells displaying cytological atypias including spindle shape or emperipolesis images [x1000, May Grunwald-Giemsa [MGG] stain]. **C**: Representative image of a multinucleated histiocyte [double arrowheads] in the bone marrow aspiration displaying emperipolesis with the cytoplasmic engulfment of two large lymphomatous cells [single arrow heads], and one small normal lymphocyte [arrow]. One of the lymphomatous cells shows a cytoplasmic engulfment of an erythrocyte [x1000, May Grunwald-Giemsa [MGG] stain]. **D**: Representative image of two histiocytic cells [x1000, May Grunwald-Giemsa [MGG] stain]. **E**: FISH analysis showing the same cells seen in Panel C : each of the two lymphomatous cells [single arrow heads] display one red signal illustrating the monosomy 13 identified on karyotype and three green signals for the centromeric 12 probes. Multiple copies of the same pattern are shown within the nuclei of the multinucleated histiocyte [double arrowheads], while the small lymphocyte is displaying a normal pattern with two green and two red signals [arrow] [x1000]. **F**: FISH analysis on the same two histiocytic cells [from panel D]: one cell shows loss of one chromosome 13 signal and three signals for chromosome 12 while the other cell exhibits a duplication of the same pattern [x1000]

**Fig. 2:**
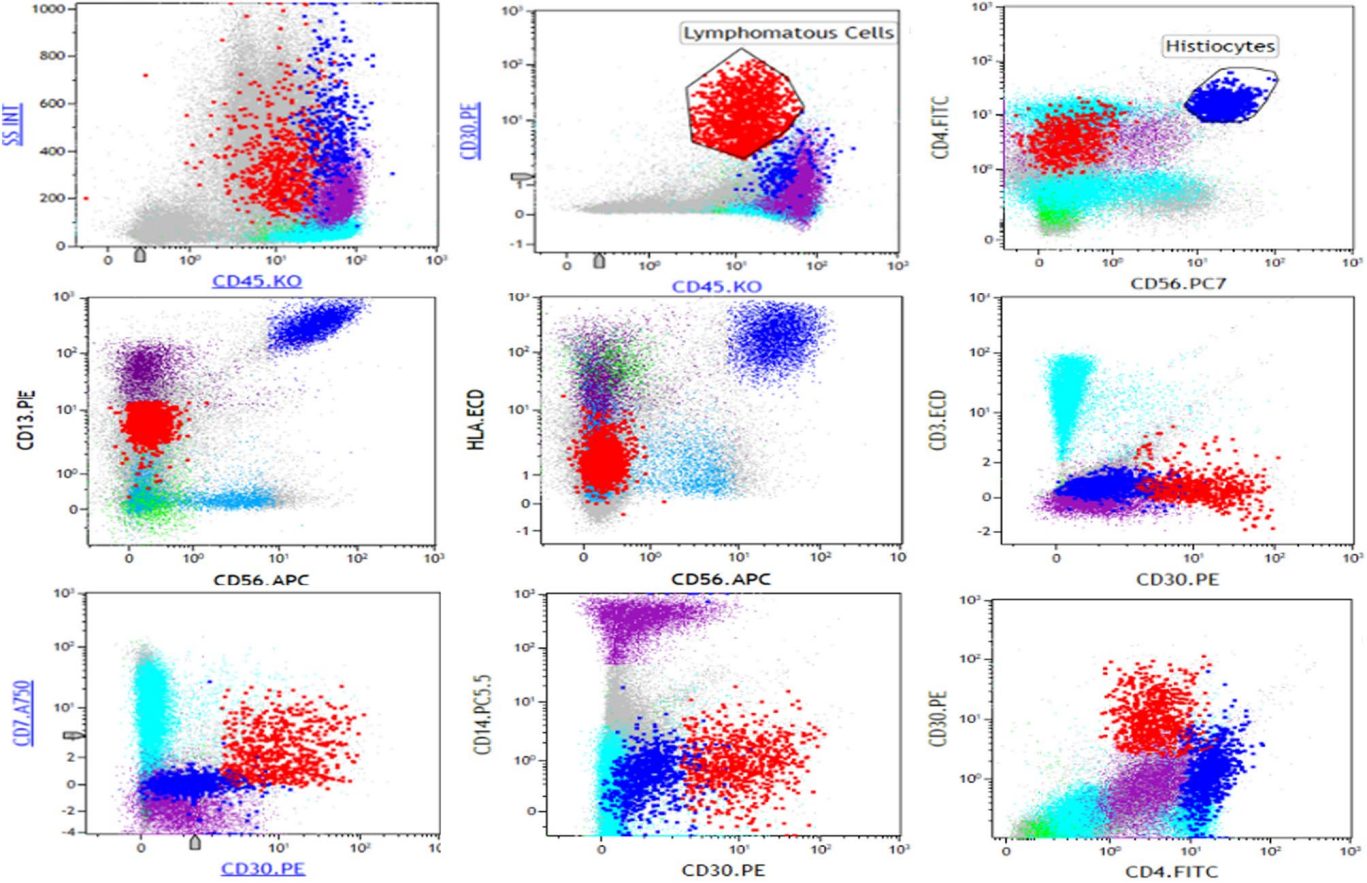
Flow cytometry plots showing lymphomatous cells in red and histiocytes in dark blue. Lymphoma cells expressed the following immunophenotype: CD30 + CD4+/- CD56- CD13- HLADR- CD3- CD7+/- CD14-. Histiocytes expressed the following immunophenotype:CD30- CD4 + + CD56 + + CD13 + + HLADR + + CD3- CD7- CD14-

**Fig. 3:**
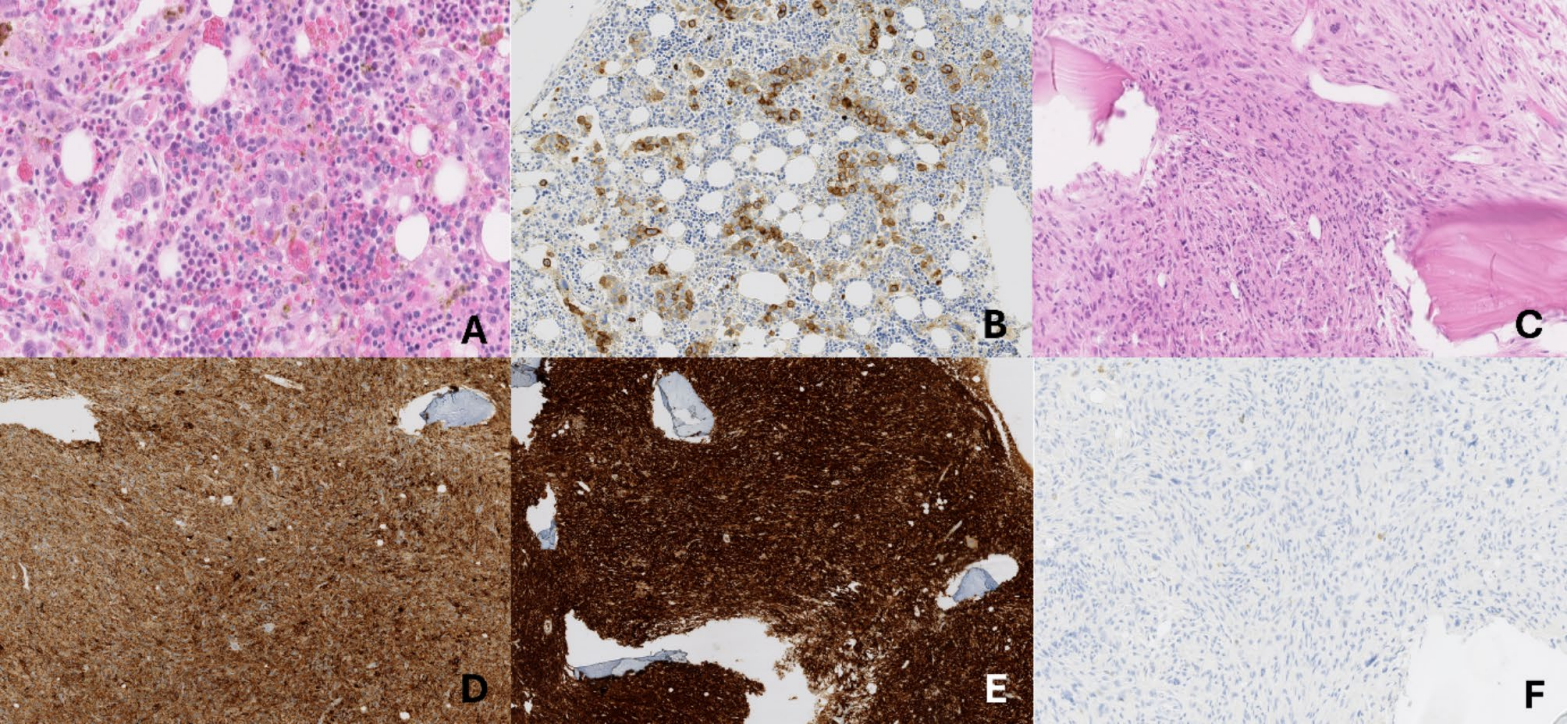
Histopathological features of the bone marrow biopsy BMB: Some areas show diffuse infiltration of the marrow by lymphoma cells with a predominant intrasinusoidal location and occasional images of emperipolesis or hemophagocytosis [hematoxylin eosin **A** [H&E], original magnification x200]. Lymphoma cells are strongly positive for CD30 **B** [Immunohistochemical [IHC] staining, original magnification x200]. Other marrow spaces show a complete replacement of the hematopoietic tissue by a neoplastic proliferation of atypical histiocytes within a fibrous stroma **C**: [H&E], original magnification x100]. Malignant histiocytes are strongly positive for CD68 **D** and CD163 **E** [IHC staining, original magnification x100]. The histiocytic area shows no CD30 expression **F** [IHC staining, original magnification x200]

PET CT-scan showed metabolic evidence in favor of high-grade lymphomatous disease [stage IV - lymph node, spleen, liver, and bone marrow involvement]. The patient underwent bone marrow aspiration [BMA] and biopsy [BMB]. BMA revealed two malignant cell proliferations: Large pleiomorphic lymphoid cells [Fig. 1A], showing partially “hallmark” cell morphology, representing 26% of the total nucleated cells, and displaying by flow cytometric analyses the following immunophenotype [CD2- CD3- CD4+/- CD5- CD7+/- CD8- CD13- CD14- CD30 + CD33- CD56- HLADR-] were associated with numerous histiocytes showing cytological atypias including gigantism, multinucleation, spindle shape, and emperipolesis images [Fig. 1B]. Histiocytes expressed an aberrant immunophenotype by flow cytometry [CD2- CD3- CD4 + + CD5- CD7- CD8- CD13 + + CD14- CD33- CD30- CD56 + + HLADR+] [Fig. 2]. Histopathological examination of the BMB [Fig. 3] mainly identifies a few areas of infiltration of the marrow by lymphoma cells with a predominant intrasinusoidal localization. Lymphoma cells strongly expressed CD30 and EMA but were negative for ALK, CD1a, CD2, CD3, PU.1, and CD68 (data not shown).They were associated with dispersed atypical histiocytes which occasionally show images of emperipolesis or hemophagocytosis. Furthermore, in several medullary spaces the hematopoietic tissue was replaced by a significant neoplastic proliferation of atypical histiocytes within a fibrous stroma and a virtual absence of the lymphomatous component. Tumor cells in these areas strongly express CD68, CD163 and PU.1 but were negative for ALK, CD30 and CD1a by immunohistochemical staining. S100 was expressed in scattered histiocytic cells [< 50% of histiocytes]. Findings were consistent with anaplastic lymphoma kinase negative anaplastic large cell lymphoma [ALK-ALCL] associated with synchronous malignant histiocytosis according to the 2016 revised classification criteria of the Histiocyte Society [1].

After cell sorting by flow cytometry, NGS was performed on different sorted cell fractions. *KRAS* c.35G > T, p.[Gly12Val] and *TP53* c.844 C > T, p.[Arg282Trp] mutations were only detected in monocytes and histiocytes while clonal TCR Gamma gene rearrangement was noted in lymphoma cells exclusively.

Cytogenetic analysis performed on the BMA revealed a complex karyotype. FISH analyses were negative for ALK and DUSP22 rearrangement, but confirmed that both malignant cell types shared, at least, monosomy 13 and the supernumerary marker chromosome identified as a partial chromosome 12 [Fig. 1C-F].

At diagnosis, the H-score was 203, indicating a risk of hemophagocytic lymphohistiocytosis of 88–93%. First-line treatment protocol included Brentuximab vedotin [1.8 mg/kg], cyclophosphamide [750 mg/m²], doxorubicin [50 mg/m²] and methylprednisolone [64 mg] [Bv-CHP]. The patient was not eligible for etoposide or MEK-inhibitor [for the clonal histiocytic component] because of his low platelets count and the high risk of hemorrhage. Two cycles were administered separated by a 3 weeks interval. Multiple complications occurred including severe encephalopathy that reversed spontaneously, septic shock with febrile neutropenia from a digestive tract infection, hemorrhagic shock due to bleeding from a femoral catheter and severe thrombocytopenia, as well as pneumonia associated with ventilation due to infection by Pseudomonas aeruginosa and Stenotrophomonas.

Hematological findings included WHO grade IV thrombocytopenia of mixed central (hemophagocytic lymphohistiocytosis, bone marrow infiltration, toxicity of chemotherapy) and peripheral origin. The patient received several lines of treatment for possible immune thrombocytopenic purpura without improvement [intravenous immunoglobulins, corticosteroids, Elthrombopag and Rituximab]. Thormbocytopenia finaly responded to Avathrombopag.

After the second cycle, the PET scan revealed a mixed response: there was partial improvement in lymph node involvement, but progression in the liver and spleen, accompanied by the emergence of a circulating clonal T lymphoma cells. His clinical condition deteriorated rapidly, leading to the onset of severe lymphohistiocytic hemophagocytosis and a swift decline, resulting in death in the intensive care unit.

## Discussion

Synchronous malignant histiocytoses were reported in association with B cell lymphomas that included mainly follicular lymphoma, chronic lymphocytic leukemia, splenic marginal zone lymphoma, mantle cell lymphoma, diffuse large-B cell lymphoma, Hodgkin lymphoma, Burkitt lymphoma and B-lymphoblastic lymphoma [2–10]. In most instances, B-cell hemopathy preceded the malignant histiocytosis. Molecular analysis of synchronous malignant histiocytosis often revealed a cytogenetic or molecular anomalies similar to that of the associated lymphomatous pathology [3]. In some cases, analysis of matched samples identified identical clonal abnormalities in both populations within the same patient, indicating a potential “transdifferentiation” during clonal evolution. In this case, the term “secondary malignant histiocytosis” can be applied. Notably, associations with follicular lymphoma frequently revealed IgH-BCL2 rearrangement in both lineages. Other identical monoclonal chromosomal and molecular aberrations found in matched samples for histiocytic and lymphomatous populations were detailed in Table 1.

**Table 1:** Identical monoclonal cytogenetic or molecular aberrations in both histiocytic and lymphomatous populations demonstrated by analysis of matched samples

Refe-rence	Hemopathie	Cytogenetic	Molecular aberration
2	FL	IgH-BCL2 rearrangement	ND
		Chromosomes 9q and 19q aberrations	CREBBP, KMT2D
	CLL	Del 17p	ND
	MCL	CCDN1-IgH	ND
3	FL	IGK gene rearrangement, BCL2 translocation	KMT2D, BCL2, POU2AF1
	T-ALL	TCR gene rearrangement, chromosome 9p aberration, CDKN2A deletion	KRAS, FBXW7
5	Transformed FL (DLBCL)	IgH gene rearrangement	ND
7	BL	t(8;14)	ND
8	B-ALL	IgH-MYC rearrangement, IGK rearrangement	35 common mutations
9	FL	IgH-BCL2 rearrangement	KMT2D, CREBBP, SPEN, PDE4DIP, ARHGAP26, PMS2, ATM, SPECC1, ZNF384

Note Patients described in references 4, 6, 10, 12, 13 and 14 did not undergo concomitant cytogenetic and/or molecular analysis of both the histiocytic and lymphomatous/leukemic populations. A clonal relationship cannot therefore be established

Moreover, certain cases reported *RAS/RAF/MEK/ERK* pathway mutations exclusively in histiocytic cells, a pathway commonly implicated in histiocytic neoplasms [2, 3, 9, 10]. These findings imply the existence of a common progenitor capable of evolving into both malignant diseases through oncogenesis involving shared and/or distinct genetic alterations [e.g., *RAS* mutation may predispose to the development of an additional clonally related hematological malignancy or a secondary malignant histiocytosis] [2, 3, 11].

Conversely, the association between malignant histiocytoses and T cell lymphomas is infrequent. Only a few cases were described in the literature and included T-cell lymphoblastic lymphoma/leukemia [3], peripheral T-cell lymphoma [3], angioimmunoblastic T-cell lymphoma [12], mycosis fungoid [13], NK/T-cell lymphoma [14]. Furthermore, anaplastic large cell lymphoma was already described in association with some clonal histiocytosis such as Langerhans cell histiocytosis or Rosai-Dorfman disease [13, 15]. To our knowledge, this is the first case of anaplastic large cell lymphoma to be reported in association with synchronous malignant histiocytosis. In this case, we succeeded in proving both the clonal nature of the malignant histiocytosis and the sharing of identical chromosomal abnormalities between the two neoplastic populations, suggesting a common precursor.

The first intriguing question related to the diagnosis was to determine whether the histiocytic population is reactive to the lymphoma clone or malignant by itself [which was never previously described in association with anaplastic large cell lymphoma]. We were able to prove the clonality of the histiocytic population by the Targeted Next Generation Sequencing [NGS] that was done on the different populations after cell sorting of the BMA sample by flow cytometry, identifying the oncogenic *KRAS*^*G12V*^ mutation that was detected exclusively in the mono-histiocytic population, proving its clonality.

The sharing of cytogenetic anomalies constituted the major argument for the existence of a common progenitor for the two malignant proliferations described in this reported case. However, neoplastic cells were distinguished by the exclusivity of the rearrangement of TCR genes within the lymphoma cells, whereas mutations in the *KRAS* and *TP53* genes selectively affected some monocytes and histiocytic cells, contrasting with the great majority of reported cases where T cell lymphoproliferative disorders and secondary/ synchronous associated malignant histiocytosis shared the same TCR gene rearrangement. Two hypotheses can be proposed regarding the presence of shared chromosomal abnormalities.

### Dedifferentiation of the lymphomatous population

Findings in our case might provide grist for the hypothesis of a possible dedifferentiation of the lymphoma cells followed by a subsequent histiocytic differentiation which would have been driven by the *KRAS*^*G12V*^. Indeed, the role of this mutation in inducing the myelomonocytic differentiation of multipotent hematopoietic stem cells was already well established [16]. On the other side, PU.1 is a transcription factor that is down regulated in T lymphoid cells and up regulated in myelomonocytic cells and macrophages. Therefore, the loss of T cell commitment of already committed T lymphoid cells and its reprogramming allowing its histio-macrophagic differentiation would also be reinforced by the over-expression of PU1, as noted in the neoplastic histiocytes [17]. This hypothesis assumes the possibility of a lymphomatous cell to dedifferentiate while retaining the cytogenetic abnormalities pre-existing to this process. However, the absence of a shared TCR rearrangement between the two cell lines casts doubt on this hypothesis.

### Chromosomal abnormalities could have emerged early in tumorigenesis, preceding the differentiation into two distinct populations

The common progenitor for both populations likely acquired its chromosomal abnormalities at an early stage and was subsequently differentiated into either a T lymphomatous cell [by rearranging its TCR gene] or into a histiocytic cell [driven by the acquirement of the oncogenic *KRAS*^*G12V*^ mutation and the increased PU.1 expression]. This hypothesis may explain the absence of the TCR rearrangement in the histiocytic lineage. The term ‘synchronous malignant histiocytosis’ is therefore more appropriate here than ‘secondary malignant histiocytosis.’

Treatment of such synchronous or secondary malignant histiocytosis frequently consists of chemo- or chemo-immunotherapy targeting the lymphomatous disease, as outlined in Table 2. In some cases, malignant histiocytosis developed several years after the initial diagnosis (metachronous malignant histiocytosis). In the absence of relapse of the primary hematologic disease, chemotherapy is the most frequently used treatment. One case reported the use of targeted therapy against the MAPK pathway [8]. Many patients died quickly after the malignant histiocytosis diagnosis.

**Table 2:** Treatment and outcome of synchronous/ metachronous malignant histiocytosis associated with primary hemopathy

Refe-rence	Case/ review	Lymphoma	Clonal relationship with MH (Table 1)	Interval between initial hemopathie and MH	Successive treatments from the MH diagnosis	Outcome from MH diagnosis
2	Case	FL	Yes	Synchronous	CIT (1st line) CT (2nd line)	Died 11 months later
	Review	Transformed FL (DLBCL)	Yes	13 years after initial FL diagnosis	CIT (1st line) CT (2nd line)	Died in the year
	Review	DLBCL	No	1 year	CIT (1st line) CT + RT (2nd line) Supportive care	Died quickly (delay?)
	Review	FL	No	8 years	Supportive care	Died 3 months later
	Review	FL	Yes	4 years	Protease inhibitor	Died 9 months later
	Review	MCL	Yes	18 months	CIT Surgery	Progression
	Review	MZL	Yes	6 years	No treatment	Died 2 months later
7	Case	BL	Yes	22 months	High intensity chemo-immunotherapy	Alive 9 years after treatment
8	Case	B-ALL	Yes	1 year	High intensity chemotherapy (1st and 2nd line) BRAFi + lenalidomide (3rd line)	Secondary MDS – died 3 years later
9	Case	FL	Yes	Few months	Radiation (1st line), Chemo-immunotherapy (2nd and 3rd line), chemotherapy + ibrutinib (4th line)	Died in the year
10	Case	SMZL	No	ND	No treatment	Died before introduction of treatment
12	Case	AITL	No	Synchronous	No treatment (declined)	Died 5 months later

In our case, the identification of a *KRAS* mutation prompted us to consider the utilization of cobimetinib or trametinib [MEK-inhibitors] in conjunction with conventional therapy as described in primary malignant histiocytosis [18, 19]. However, the extreme rarity of secondary malignant histiocytosis and the limited literature on the association of anti-MEK therapy in this context prevented us from establishing a consensual approach. Nevertheless, due to the presence of severe and refractory thrombocytopenia, the addition of this treatment was inappropriate given the increased risk of haemorrhage previously reported in such a context [20].

## Conclusion

Histiocytosis associated with lymphoproliferative disorders are mostly reactive. Clonally related malignant histiocytosis sharing mutations and/or cytogenetic anomalies with the lymphoma cells, are rare and frequently associated with mutations affecting genes of the RAS/MAPK pathway. The absence of TCR rearrangement in the histiocytes of our patient strongly suggests that chromosomal abnormalities were acquired early in the differentiation process of the common progenitor, prior to their differentiation into lymphomatous or histiocytic cells. However, it is also plausible that the lymphomatous cells underwent dedifferentiation and subsequently differentiated into histiocytic cells. Identification of a mutation in the MAPK pathway would justify the use of therapy targeting such a pathway in the absence of any counter-indication forbidding this treatment.

## Data Availability

No datasets were generated or analysed during the current study.

## Abbreviations

ALK-ALCL: Anaplastic lymphoma kinase negative anaplastic large cell lymphoma
BMA: Bone marrow aspiration
BMB: Bone marrow biopsy
FISH: Fluorescence in situ hybridization
NGS: Next Generation Sequencing
RV: Reference values
